# Hypertriglyceridemic waist phenotype: Association with initial neurological severity and etiologic subtypes in patients with acute ischemic stroke

**DOI:** 10.3389/fendo.2022.1024398

**Published:** 2022-12-01

**Authors:** Yuan Ren, Zi-Han Qiu, Wei-Hua Wu, Xiao-Guang Dong, Shuang Han, Fu-Liang Zhang, Fan-Li Kong, Feng-E Li

**Affiliations:** ^1^ Department of Postgraduate, School of Clinical Medicine, Beihua University, Jilin, China; ^2^ Department of Neurology, Zhujiang Hospital, Southern Medical University, Guangzhou, China; ^3^ Department of Neurology, the Affiliated Hospital of Beihua University, Jilin, China; ^4^ Department of Neurology, The First Hospital of Jilin University, Changchun, China; ^5^ Department of Pathophysiology, School of Basic Medicine, Beihua University, Jilin, China

**Keywords:** waist circumference, ischemic stroke, hypertriglyceridemia, severity, etiology

## Abstract

**Objective:**

To explore the relationship of hypertriglyceridemic waist phenotype (HTWP) with initial neurological severity and etiologic subtypes in patients with acute ischemic stroke.

**Methods:**

The data for this study were collected from hospitalized patients within 72 h of acute ischemic stroke onset at the Department of Neurology of the Affiliated Hospital of Beihua University from 1 July 2020 to 30 June 2022. The initial neurological severity was assessed by the National Institute of Health Stroke Scale (NIHSS) on the day of admission: NIHSS <6 was defined as mild stroke, and NIHSS ≥6 as moderate to severe stroke. HTWP was defined by fasting serum triglycerides ≥1.7 mmol/L and waist circumference ≥90 cm in men and ≥80 cm in women. Differentiation of etiologic subtypes was based on the method reported in the Trial of Org 10 172 in Acute Stroke Treatment. Multivariate logistic regression analysis was used to analyze the association of HTWP with initial neurological severity and etiologic subtypes.

**Results:**

The study included 431 patients. Compared with the normal waist–normal blood triglyceride group, patients with HTWP had reduced risks of moderate to severe stroke [odds ratio (OR): 0.384, 95% confidence interval (CI): 0.170–0.869; *P* = 0.022]. In addition, the risk of small-artery occlusion stroke was 2.318 times higher in the HTWP group than in the normal triglyceride–normal waist (NWNT) group (OR: 2.318, 95% CI: 1.244–4.319; *P* = 0.008).

**Conclusion:**

Initial neurological severity was less severe in patients with HTWP, and HTWP was associated with an increased risk of small-artery occlusion stroke.

## Introduction

Stroke is a disease with high morbidity and mortality worldwide and is a major contributor to disability (1). With the rapid development of China’s economy and the improvement in people’s living standards, exposures to some cerebrovascular risk factors like smoking, overweight or obesity, hypertension, and diabetes mellitus are on the rise; the incidence of stroke has continued to increase over the past 30 years, and China faces the highest burden of stroke in the world (2, 3). Ischemic stroke is the most common type of cerebrovascular disease, constituting 69.6% and 77.8% of stroke incidence and prevalence, respectively, in China (3). Although revolutionary progress has been made by intravenous thrombolysis and intravascular therapy in hyperacute cerebral infarction in recent years (4, 5), substantial challenges remain to enhance the curative effect of traditional medicine therapy when patients miss the thrombolytic time window, especially those patients with disorders of motor function and activities of day-to-day living, which carry enormous consequences for societies and economies (6). Thus, controlling risk factors, early diagnosis, accurate assessment of initial neurological severity, and appropriate treatment are of great significance for the prognosis of ischemic stroke.

Though obesity is an independent risk factor for ischemic stroke, numerous studies have shown an inverse association between obesity and clinical prognosis in patients with ischemic stroke, and this phenomenon is known as the obesity paradox (7–11). However, obesity does not fully account for the influence of body fat distribution, and further studies found that waist circumference (WC) could more accurately reflect the accumulation of visceral fat and the degree of atherosclerosis; thus, it was more strongly related to ischemic stroke than body mass index (BMI) (12, 13). The Northern Manhattan Stroke Study indicated that abdominal obesity is an independent, potent risk factor for ischemic stroke (12), but higher WC has been linked to milder baseline stroke severity and better functional outcomes among patients with acute ischemic stroke (14, 15). Apart from obesity, a prospective cohort study showed that triglycerides (TGs) are positively associated with ischemic stroke (16). In contrast, clinical data from retrospective observational studies suggest that patients with higher TG also manifest milder neurological severity and better early outcomes (17, 18). Based on the above research findings, it seemed that an obesity paradox existed in ischemic stroke despite different obesity measures. However, recent studies found that the obesity paradox does not apply to all individuals, and whether it exists is affected by numerous factors such as sex, uric acid, and insulin sensitivity (19–21). Importantly, a large sample size retrospective study found that obesity was not associated with the risk of death in the first month after stroke, and patients with higher BMI had a stroke at a younger age, suggesting that the obesity paradox in stroke is not real and may be caused by selection bias (22).

Currently, only computed tomography (CT) and magnetic resonance imaging (MRI) can accurately measure the content of visceral fat (23–25), but these imaging techniques are not suitable for health screening in large populations because of the high cost and radiation exposure. Since the measurement of WC alone is ineffective in distinguishing between subcutaneous and visceral adipose tissues (26), the concept of hypertriglyceridemic waist phenotype (HTWP) (defined as coexisting hypertriglyceridemia and an elevated WC) was put forward by Lemieux et al. (27) to solve the abovementioned problem, which was used as a simple marker to identify individuals with metabolic abnormalities and increased visceral fat (28–30). To date, two prospective studies have shown that HTWP is associated with an increased risk of ischemic stroke (31, 32). Nevertheless, there is no research on the association between HTWP and the clinical manifestation of acute ischemic stroke. Hence, this retrospective cohort study aimed to evaluate the effects of the HTWP on initial neurological severity and etiologic subtypes in patients with acute ischemic stroke.

## Materials and methods

### Inclusion and exclusion criteria

The data for this study were collected from consecutive hospitalized patients at the Department of Neurology of the Affiliated Hospital of Beihua University from 1 July 2020 to 30 June 2022. The research proposal was approved by the Medical Ethics Committee of the Affiliated Hospital of Beihua University (2021-R-17). The inclusion criteria included the following: 1) in line with the Chinese 2018 guidelines for the early management of patients with acute ischemic stroke (33), all patients were diagnosed by brain CT and MRI examinations; 2) the study sample consists of consecutive first-ever acute ischemic stroke patients with an onset ≤72 h; 3) the patients and their families were aware of the study and signed a consent form; and 4) complete and detailed clinical data sets were available. The exclusion criteria were as follows: 1) CT or MRI examination of the brain indicating cerebral hemorrhage or non-acute vascular brain lesions, 2) previous history of stroke with neurological impairment, 3) a history of taking lipid-lowering drugs 1 month before the investigation, and 4) transient ischemic attacks without ischemic lesions visible on MRI performed within 24 h of stroke onset.

### Data collection

According to the World Health Organization standardized protocols, trained professionals completed physical examinations (e.g., resting blood pressure, WC) on admission. Blood pressure was measured by an electronic sphygmomanometer (OMRON HEM-7211), and each patient was measured two consecutive times at an interval of 2 min while resting for at least 15 min before, and the average was taken as the final result. WC was measured with a tapeline to the nearest 0.1 cm at the midpoint between the lower margin costal arch and anterior superior iliac spine at the end of expiration (34). For partially paralyzed and bedridden patients with acute ischemic stroke, their WC was measured at the level of the umbilicus using a measuring tape (14). All patients underwent diagnostic tests, including routine and biochemical blood tests, brain CT and MRI scans, and cerebrovascular, cervical vascular, and cardiac ultrasound.

### Definitions of the HTWP and other phenotypes

According to the criteria for metabolic syndrome in the Chinese population established by the International Diabetes Federation, hypertriglyceridemia was defined as TG ≥1.7 mmol/L and abdominal obesity as WC ≥90 cm for men and ≥80 cm for women (34). Patients were divided into three groups: 1) normal waist–normal blood TG (NWNT): TG <1.7 mmol/L and WC <90 cm (men) or <80 cm (women); 2) elevated waist–normal blood TG (EWNT)/normal waist–elevated blood TG (NWET): TG <1.7 mmol/L and WC ≥90 cm (men) or ≥80 cm (women)/TG <1.7 mmol/L and WC ≥90 cm (men) or ≥80 cm (women); and 3) HTWP: TG ≥1.7 mmol/L and WC ≥90 cm (men) or ≥80 cm (women).

### Diagnostic criteria for initial neurological severity and etiologic subtypes

The National Institute of Health Stroke Scale (NIHSS) was independently assessed by two neurologists on the day of admission, and the consistency test was conducted to assess the results. NIHSS on admission was used as the main indicator to evaluate the initial neurological severity of an acute ischemic stroke, categorized as mild (0–5), moderate (6–13), or severe (≥14) (35). According to clinical manifestations, imaging, and laboratory examinations, the patients were divided into four etiologic subtypes based on the method reported in the Trial of Org 10 172 in Acute Stroke Treatment (36, 37): 1) large-artery atherosclerosis (LAA), 2) small-artery occlusion (SAO), 3) cardioembolism (CE), and 4) stroke of other determined etiology and undetermined etiology (SOE and SUE).

### Diagnostic criteria of risk factors for stroke

Smokers indicated continuous or accumulative smoking for 6 months or more (38). Alcohol drinkers reported taking more than 14 standard drinks per week for men and seven standard drinks per week for women, following the National Institute on Alcohol Abuse and Alcoholism guidelines (39). Hypertension was designated as systolic blood pressure (SBP) ≥140 mmHg, diastolic blood pressure (DBP) ≥90 mmHg, or taking antihypertensive therapy (40). Diabetes was considered fasting blood glucose at least at the level of 7.0 mmol/L or taking hypoglycemic therapy (41). Atrial fibrillation and coronary heart disease (CHD) were based on a self-reported history and the results of an electrocardiogram at admission. Anemia was defined as hemoglobin <130 g/L in men and <120 g/L in women or those taking anti-anemia therapy (42). Physical activity was defined as the performance of heavy physical labor or regular physical exercise for more than 1 year, more than three times per week, and for at least 30 min per session.

### Statistical method

SPSS 19.0 statistical software (SPSS Inc., Chicago, IL, USA) was used for the statistical analysis. The Shapiro–Wilk normality test was performed based on the data obtained. Non-normal distribution data were expressed as median and quartile range. Differences between continuous variables were compared using the Mann–Whitney test (two groups) or the Kruskal–Wallis test (multiple groups). Enumeration data were expressed as frequency and percentage, and comparisons between groups were performed using the *χ* ^2^ test. Because atrial fibrillation occurs only in CE and SUE, atrial fibrillation was not included in the stroke subtype model. Multivariate logistic regression analysis was used to identify independent risk factors, and the odds ratio (OR) and 95% confidence interval (CI) were calculated. *P <*0.05 was considered statistically significant.

## Results

**Figure 1:**
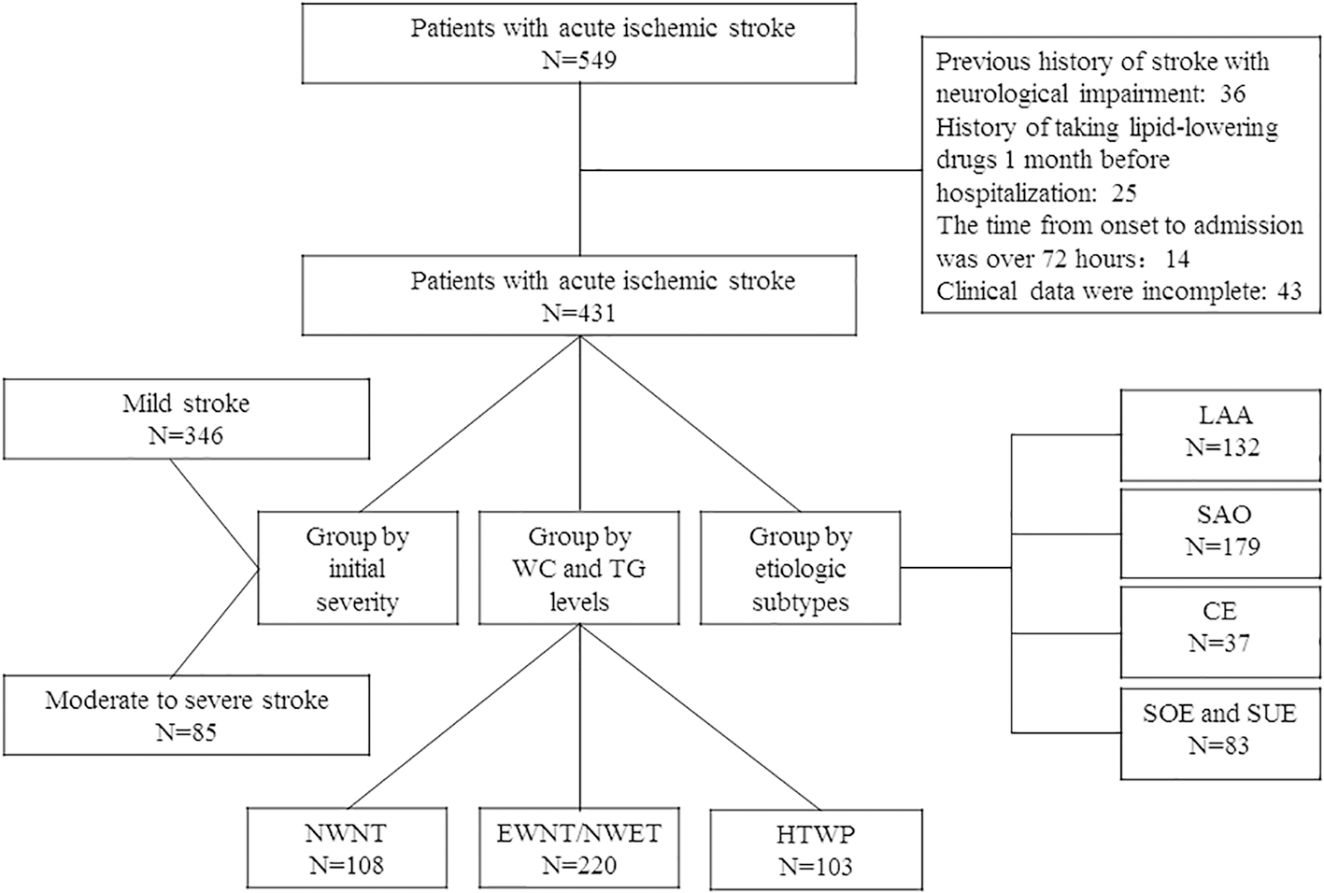
Flowchart of the research object selection.

From 1 July 2020 to 30 June 2022, 549 hospitalized patients within 72 h of stroke onset were included in this study; after screening, 431 of them met the inclusion criteria, as shown in the flowchart (**Figure 1**). The patients’ demographic and clinical characteristics are shown in **Table 1**. The median patient age was 64 (interquartile range: 58–72) years, and 67.05% were men. Among the 431 patients included, 346 (80.28%) had mild stroke, and 85 (19.72%) had moderate or severe stroke on admission. Compared with patients with mild stroke, patients with moderate to severe stroke at admission had higher rates of atrial fibrillation (*P* < 0.001). In contrast, patients with moderate to severe stroke at admission had less physical activity (*P* = 0.003).

**Table 1:** Characteristics of the patients stratified by initial neurological severity.

Variables	Total	Mild stroke	Moderate to severe stroke	*P-*value
	*N* = 431	*N* = 346	*N* = 85	
Age (years)	64.0 (58.0–72.0)	64.5 (57.0–71.0)	64.0 (58.0–75.5)	0.337
Gender (male)	289 (67.05)	234 (67.63)	55 (64.71)	0.607
Residence (rural)	131 (30.39)	105 (30.35)	26 (30.59)	0.965
Educational level (≥ high school)	131 (30.39)	109 (31.50)	22 (25.88)	0.313
Hypertension	331 (76.80)	263 (76.01)	68 (80.00)	0.435
Diabetes	151 (35.03)	118 (34.10)	33 (38.82)	0.414
Atrial fibrillation	30( 6.96)	16 (4.62)	14 (16.47)	<0.001
Coronary heart disease	100 (23.20)	79 (22.83)	21 (24.71)	0.714
Anemia	39 (9.05)	31 (8.96)	8 (9.41)	0.896
Smoker	212 (49.19)	167 (48.27)	45 (52.94)	0.44
Alcohol drinker	141 (32.71)	117 (33.82)	24 (28.24)	0.326
Physical activity	250 (58.00)	230 (66.47)	42 (49.41)	0.003
Etiologic subtypes				<0.001
LAA	132 (30.63)	101 (29.19)	31 (36.47)	
SAO	179 (41.53)	167 (48.27)	12 (14.12)	
CE	37 (8.58)	24 (6.94)	13 (15.29)	
SOE and SUE	83 (19.26)	54 (15.61)	29 (34.12)	
WC and TG data				0.030
NWNT	108 (25.06)	78 (22.54)	30 (27.78)	
EWNT/NWET	220 (51.04)	179 (51.73)	41 (18.64)	
HTWP	103 (23.90)	89 (25.72)	14 (16.47)	

Values are expressed as median (Q1–Q3) or n (%).

LAA, large-artery atherosclerosis; SAO, small-artery occlusion; CE, cardioembolism; SOE and SUE, stroke of other determined etiology and undetermined etiology; NWNT, normal waist–normal blood triglycerides; EWNT, elevated waist–normal blood triglycerides; NWET, normal waist–elevated blood triglycerides; HTWP, hypertriglyceridemic waist phenotype.

As shown in **Table 2**, patients were assigned to three groups according to their WC and TG data. There were 108, 163, and 103 patients in the NWNT, EWNT/NWET, and HTWP groups, respectively. Age, the proportion of hypertension, diabetes, CHD, initial neurological severity, and etiology types showed statistically significant differences among the three groups (*P* < 0.05). Compared with the NWNT group, the proportion of hypertension, diabetes, and CHD in the HTWP group was higher (*P* < 0.05), while age was lower (*P* < 0.05).

**Table 2:** Characteristics of the patients stratified by WC and TG data.

Variables	NWNT	EWNT/NWET	HTWP	*P-*value
	*N* = 108	*N* = 220	*N* = 103	
Age (years)	66.0 (59.3–74.0)	65.0 (57.0–71.8)	63.0 (56.0–68.0)^*^	0.007
Gender (male)	80 (74.07)	147 (66.82)	62 (60.19)	0.100
Residence (rural)	36 (33.33)	65 (29.55)	30 (29.13)	0.743
Educational level (≥ high school)	43 (39.81)	68 (30.91)	30 (29.13)	0.183
Hypertension	81 (75.00)	159 (72.27)	91 (88.35)^*^	0.005
Diabetes	18 (16.67)	78 (35.45)^*^	55 (53.40)^*^	<0.001
Atrial fibrillation	10 (9.26)	18 (8.18)	2 (1.94)	0.067
Coronary heart disease	25 (23.15)	42 (19.09)	33 (32.04)^*^	0.037
Anemia	14 (12.96)	20 (9.09)	5 (4.85)	0.184
Smoker	58 (53.70)	106 (48.18)	48 (46.60)	0.536
Alcohol drinker	35 (32.41)	75 (34.09)	31 (30.10)	0.773
Physical activity	71 (65.74)	140 (63.64)	61 (59.22)	0.602
Initial neurological severity				0.030
Mild stroke	78 (22.54)	179 (51.34)	89 (25.72)	
Moderate to severe stroke	30 (27.78)	41 (18.64)	14 (16.47)	
Etiologic subtypes				0.009
LAA	40 (37.04)	62 (28.18)	30 (29.13)	
SAO	31 (28.70)	97 (44.09)	51 (49.51)	
CE	16 (14.81)	18 (8.18)	3 (2.91)	
SOE and SUE	21 (19.44)	43 (19.55)	19 (18.45)	

Values are expressed as median (Q1–Q3) or n (%).

LAA, large-artery atherosclerosis; SAO, small-artery occlusion; CE, cardioembolism; SOE and SUE, stroke of other determined etiology and undetermined etiology; NWNT, normal waist–normal blood triglycerides; EWNT, elevated waist–normal blood triglycerides; NWET, normal waist–elevated blood triglycerides; HTWP, hypertriglyceridemic waist phenotype.

^*^P < 0.05 compared with NWNT.

As shown in **Table 3**, compared with patients with NWNT, single-factor logistic regression analysis showed that patients with HTWP had reduced risks of moderate to severe stroke (OR: 0.409, 95% CI: 0.202–0.826; *P* = 0.013, **Table 3**). Furthermore, the association of WC and TG data with initial neurological severity was assessed by three different multivariate logistic regression analysis models. After adjusting for various potential confounding factors, including age, sex, residence and education levels, hypertension, diabetes, atrial fibrillation, CHD, anemia, smoking, alcohol consumption, physical activity, and stroke etiologic subtypes, this pattern of significant association remained (OR: 0.384, 95% CI: 0.170–0.869; P = 0.022, **Table 3**).

**Table 3:** Odds ratio (95% confidence interval) for moderate to severe stroke according to WC and TG data.

	NWNT	NWET/EWNT	HTWP
Unadjusted	1.000 (Ref)	0.596 (0.347–1.023)	0.409 (0.202–0.826)
*P*-value		0.060	0.013
Model 1	1.000 (Ref)	0.591 (0.341–1.024)	0.401 (0.195–0.826)
*P*-value		0.061	0.013
Model 2	1.000 (Ref)	0.540 (0.301–0.969)	0.358 (0.162–0.789)
*P*-value		0.039	0.011
Model 3	1.000 (Ref)	0.555 (0.300–1.028)	0.384 (0.170–0.869)
*P*-value		0.061	0.022

Model 1: adjusted for age, gender, residence, and education level; model 2: adjusted for model 1, hypertension, diabetes, atrial fibrillation, coronary heart disease, anemia, smoker, alcohol drinker, and physical activity; model 3: adjusted for model 2 and stroke etiologic subtypes.

As shown in **Table 4**, all patients were divided into four groups according to stroke etiologic subtypes, including 132, 179, 37, and 83 patients in the LAA, SAO, CE, and SOE and SUE groups, respectively. The stroke etiologic subtypes differed by age, the proportion of CHD and anemia, initial neurological severity, and WC and TG data. Compared with patients with NWNT, multivariate logistic regression analysis showed that patients with HTWP had increased risks of SAO (OR: 2.318, 95% CI: 1.244–4.319; *P* = 0.008, **Table 5**) and reduced risks of CE (OR: 0.131, 95% CI: 0.033–0.528; *P* = 0.004, **Table 5**) after adjusting for various potential confounding factors such as age, sex, residence and education levels, hypertension, diabetes, CHD, anemia, smoking, alcohol drinking, and physical activity.

**Table 4:** Characteristics of the patients stratified by stroke etiologic subtypes.

Variables	LAA	SAO	CE	SOE and SUE	*P*-value
	*N* = 132	*N* = 179	*N* = 37	*N* = 83	
Age (years)	65.5 (59.0–74.5)	63.0 (56.5–69.0)	68.0 (60.0–77.0)	63.0 (57.0–71.0)	0.008
Gender (male)	93 (70.45)	119 (66.48)	23 (62.16)	54 (65.06)	0.737
Residence (rural)	38 (28.79)	53 (29.61)	11 (29.73)	29 (34.94)	0.794
Educational level (≥ high school)	36 (27.27)	56 (31.28)	11 (29.73)	28 (33.73)	0.772
Hypertension	108 (81.82)	135 (75.42)	30 (81.08)	58 (69.88)	0.198
Diabetes	48 (36.36)	65 (36.31)	11 (29.73)	27 (32.53)	0.821
Coronary heart disease	30 (22.73)	29 (16.20)	24 (64.86)	17 (20.48)	<0.001
Anemia	16 (12.12)	5 (2.79)	8 (21.62)	10 (12.05)	<0.001
Smoker	66 (50.00)	88 (49.16)	17 (45.95)	41 (49.40)	0.979
Alcohol drinker	45 (34.09)	64 (35.75)	9 (24.32)	23 (27.71)	0.393
Physical activity	81 (61.36)	122 (68.16)	23 (62.16)	46 (55.42)	0.235
Initial neurological severity					<0.001
Mild stroke	101 (76.52)	167 (93.30)	24 (64.86)	54 (65.06)	
Moderate to severe stroke	31 (23.48)	12 (6.70)	13 (35.14)	29 (34.94)	
WC and TG data					0.009
NWNT	40 (30.30)	31 (17.32)	16 (43.24)	21 (25.30)	
EWNT/NWET	62 (46.97)	97 (54.19)	18 (48.65)	43 (51.81)	
HTWP	30 (22.73)	51 (28.49)	3 (8.11)	19 (22.89)	

Values are expressed as median (Q1–Q3) or n (%). LAA, large-artery atherosclerosis; SAO, small-artery occlusion; CE, cardioembolism; SOE and SUE, stroke of other determined etiology and undetermined etiology; NWNT, normal waist–normal blood triglycerides; EWNT, elevated waist–normal blood triglycerides; NWET, normal waist–elevated blood triglycerides; HTWP, hypertriglyceridemic waist phenotype.

**Table 5:** Odds ratio (95% confidence interval) for stroke etiologic subtypes according to WC and TG data.

	NWNT	NWET/EWNT	HTWP
LAA			
Unadjusted	1.000 (Ref)	0.667 (0.409–1.087)	0.699 (0.392–1.244)
*P*-value		0.104	0.223
Model 1	1.000 (Ref)	0.715 (0.435–1.175)	0.792 (0.437–1.436)
*P*-value		0.186	0.443
Model 2	1.000 (Ref)	0.696 (0.417–1.162)	0.719 (0.384–1.346)
*P*-value		0.166	0.302
SAO			
Unadjusted	1.000 (Ref)	1.959 (1.195–3.212)	2.436 (1.380–4.301)
*P*-value		0.008	0.002
Model 1	1.000 (Ref)	1.186 (1.119–3.046)	2.200 (1.231–3.933)
*P*-value		0.016	0.008
Model 2	1.000 (Ref)	1.779 (1.057–2.994)	2.318 (1.244–4.319)
*P*-value		0.030	0.008
CE			
Unadjusted	1.000 (Ref)	0.512 (0.250–1.050)	0.173 (0.049–0.611)
*P*-value		0.068	0.006
Model 1	1.000 (Ref)	0.525 (0.253–1.088)	0.181 (0.050–0.655)
*P*-value		0.083	0.009
Model 2	1.000 (Ref)	0.622 (0.273–1.415)	0.131 (0.033–0.528)
*P*-value		0.257	0.004

Model 1: adjusted for age, gender, residence, and education level; model 2: adjusted for model 1, hypertension, diabetes, coronary heart disease, anemia, smoker, alcohol drinker, and physical activity.

## Discussion

To our best knowledge, this is the first study to evaluate the association of HTWP with the clinical manifestation of acute ischemic stroke. In this study, we found that patients with HTWP had a reduced risk of moderate-to-severe stroke at admission. We also found that the etiological subtype of stroke in patients with HTWP was more likely to be SAO.

In recent years, epidemiologic studies have shown that HTWP is an independent risk factor for ischemic stroke and could be used as a simple tool to screen individuals with a high risk for ischemic stroke (31, 32). Data from a large prospective cohort study of 95,015 participants in the Kailuan community in Tangshan, China, indicated that HTWP had an unadjusted hazard ratio (HR) of 1.75 (95% CI: 1.48–2.06) for future ischemic stroke, and the HR remained significant (HR: 1.23, 95% CI: 1.01–1.49) after adjustment for confounders (31). In addition, a prospective cohort study that surveyed 4,081 participants over 35 years of age without a stroke history showed that HTWP was significantly associated with an increased risk of ischemic stroke before and after adjustment for confounding factors; the HR and 95% CI were 1.94 (1.27–2.96) and 1.71 (1.05–2.78), respectively (32).

Although no studies have evaluated the impact of HTWP on initial neurological severity, a series of studies have shown that lower TG levels and smaller WC are associated with more severe stroke (14, 15, 43, 44). Weir et al. found that lower TG levels were associated with more severe initial neurological impairment and higher mortality following acute stroke (43). Similarly, Tziomalos et al. reported that lower TG levels are associated with more severe stroke and appear to predict in-hospital mortality in patients with acute ischemic stroke (44). Moreover, Kang et al. found that higher WC at admission was associated with milder baseline stroke severity and better functional outcomes following acute ischemic stroke (14, 15). In line with the above research findings, our data showed that patients with HTWP were more likely to have a mild stroke. In our view, part of the reason for the above results might be linked to the difference in patients’ socioeconomic status. In China, individuals with higher family incomes tend to be overweight or slightly obese and are more likely to receive better treatment and secondary prevention after an ischemic stroke (45). Another important reason might be that individuals with HTWP are more likely to have hypertension, diabetes, and ischemic heart disease (46, 47), and they will often use prophylactic drugs to effectively control these stroke risk factors and reduce the probability of experiencing moderate to severe ischemic stroke.

Notably, studies have shown that visceral fat accumulation is associated with an increased risk of small vascular disease and lacunar infarction (48, 49). In addition, Pinto et al. found that patients with CE and SUE had greater initial neurological severity on admission and the worst prognosis either in terms of disability or mortality, while those with SAO had the least initial neurological severity at admission and the best prognosis (50). Our results suggest that HTWP was associated with SAO; therefore, we speculate that the reason why patients with HTWP were more prone to mild stroke might be related to lacunar infarction. Lemieux et al. revealed that individuals with HTWP exhibited the atherogenic metabolic triad (hyperinsulinemia, elevated apo B, and small-dense low-density lipoprotein), further leading to the development of early atherosclerosis and an increased risk of CHD (27). Furthermore, Chen et al. also found that individuals with HTWP presented higher serum uric acid levels, suggesting that hyperuricemia could be a pathophysiologic link between HTWP and atherothrombosis (51). It is worth noting that some studies have shown that insulin resistance and hyperuricemia are independent risk factors for lacunar infarction (52, 53). Thus, we speculate that patients with HTWP have insulin resistance and hyperuricemia and are more prone to lacunar stroke, but the specific mechanism needs to be further studied.

It is difficult to measure the weight of patients with acute ischemic stroke because many hemiplegic or unconscious patients cannot stand on the scale without assistance. By measuring WC and serum fasting TG, we can easily and quickly assess the patients’ visceral fat status. Therefore, HTWP can be a simple measure of visceral fat in patients with acute ischemic stroke. There are a few limitations to this study. Firstly, due to the retrospective design of this study, a causal association between HTWP and initial neurological severity in patients with acute ischemic stroke cannot be inferred. Moreover, we did not follow up on stroke patients over a long period, and the relationship between HTWP and long-term outcomes in patients with acute ischemic stroke is unclear. Additionally, our study group included only Chinese patients, and our findings may not apply to other ethnic groups.

## Data availability statement

The original contributions presented in the study are included in the article/Supplementary Material. Further inquiries can be directed to the corresponding authors.

## Ethics statement

The studies involving human participants were reviewed and approved by the Medical Ethics Committee of The Affiliated Hospital of Beihua University. The patients/participants provided their written informed consent to participate in this study.

## Author contributions

All the authors participated sufficiently in the work and approved the final version of the article. YR, F-LK and F-EL designed the study. YR and F-LZ developed the methodology. YR, Z-HQ, W-HW, X-GD and SH collected the data. YR performed the analysis and wrote the article.

## Funding

This study was supported by The Scientific Research Project of the Education Department of Jilin Province (JJKH20210064KJ) to F-EL and The Scientific Research Project of the Education Department of Jilin Province(JJKH20210053KJ) to F-LK.

## Acknowledgments

We would like to acknowledge all of the study participants and interviewers from the Affiliated Hospital of Beihua University. We gratefully acknowledge the support of Department of Education of Jilin Province, China.

## Conflict of interest

The authors declare that the research was conducted in the absence of any commercial or financial relationships that could be construed as a potential conflict of interest.

## Publisher’s note

All claims expressed in this article are solely those of the authors and do not necessarily represent those of their affiliated organizations, or those of the publisher, the editors and the reviewers. Any product that may be evaluated in this article, or claim that may be made by its manufacturer, is not guaranteed or endorsed by the publisher.

## References

[1] Roth GA Mensah GA Johnson CO Addolorato G Ammirati E Baddour LM. Global burden of cardiovascular diseases and risk factors, 1990-2019: Update from the GBD 2019 study. J Am Coll Cardiol (2020) 76:2982–3021. doi: 10.1016/j.jacc.2020.11.010 33309175 PMC7755038

[2] Zhou M Wang H Zeng X Yin P Zhu J Chen W. Mortality, morbidity, and risk factors in China and its provinces, 1990-2017: a systematic analysis for the global burden of disease study 2017. Lancet (2019) 394:1145–58. doi: 10.1016/S0140-6736(19)30427-1 PMC6891889 31248666

[3] Wang W Jiang B Sun H Ru X Sun D Wang L. Prevalence, incidence, and mortality of stroke in China: Results from a nationwide population-based survey of 480 687 adults. Circulation (2017) 135:759–71. doi: 10.1161/CIRCULATIONAHA.116.025250 28052979

[4] Wardlaw JM Murray V Berge E del Zoppo GJ. Thrombolysis for acute ischaemic stroke. Cochrane Database Syst Rev (2014) 7:CD000213. doi: 10.1002/14651858.CD000213.pub3 PMC4153726 25072528

[5] Goyal M Menon BK van Zwam WH Dippel DW Mitchell PJ Demchuk AM. Endovascular thrombectomy after large-vessel ischaemic stroke: a meta-analysis of individual patient data from five randomised trials. Lancet (2016) 387:1723–31. doi: 10.1016/S0140-6736(16)00163-X 26898852

[6] Campbell BCV De Silva DA Macleod MR Coutts SB Schwamm LH Davis SM. Ischaemic stroke. Nat Rev Dis Primers (2019) 5:70. doi: 10.1038/s41572-019-0118-8 31601801

[7] Kim Y Kim CK Jung S Yoon BW Lee SH. Obesity-stroke paradox and initial neurological severity. J Neurol Neurosurg Psychiatry (2015) 86:743–7. doi: 10.1136/jnnp-2014-308664 25209415

[8] Ovbiagele B Bath PM Cotton D Vinisko R Diener HC. Obesity and recurrent vascular risk after a recent ischemic stroke. Stroke (2011) 42:3397–402. doi: 10.1161/STROKEAHA.111.624957 21960576

[9] Vemmos K Ntaios G Spengos K Savvari P Vemmou A Pappa T. Association between obesity and mortality after acute first-ever stroke: the obesity-stroke paradox. Stroke (2011) 42:30–6. doi: 10.1161/STROKEAHA.110.593434 21127299

[10] Wohlfahrt P Lopez-Jimenez F Krajcoviechova A Jozifova M Mayer O Vanek J. The obesity paradox and survivors of ischemic stroke. J Stroke Cerebrovasc Dis (2015) 24:1443–50. doi: 10.1016/j.jstrokecerebrovasdis.2015.03.008 25866318

[11] Zhang P Yan XL Qu Y Guo ZN Yang Y. Association between abnormal body weight and stroke outcome: A meta-analysis and systematic review. Eur J Neurol (2021) 28:2552–64. doi: 10.1111/ene.14881 33896081

[12] Suk SH Sacco RL Boden-Albala B Cheun JF Pittman JG Elkind MS. Abdominal obesity and risk of ischemic stroke: The northern Manhattan stroke study. Stroke (2003) 34:1586–92. doi: 10.1161/01.STR.0000075294.98582.2F 12775882

[13] Winter Y Rohrmann S Linseisen J Lanczik O Ringleb PA Hebebrand J. Contribution of obesity and abdominal fat mass to risk of stroke and transient ischemic attacks. Stroke (2008) 39:3145–51. doi: 10.1161/STROKEAHA.108.523001 18703800

[14] Kang K Lee WW Lee JJ Park JM Kwon O Kim BK. Association of higher waist circumference with milder stroke severity in acute ischaemic stroke. Neurol Res (2018) 40:785–94. doi: 10.1080/01616412.2018.1479346 29856277

[15] Kang K Lee WW Lee JJ Park JM Kwon O Kim BK. Comparison of body mass index, waist circumference, and waist-height ratio in predicting functional outcome following ischemic stroke. J Thromb Thrombolysis (2017) 44:238–44. doi: 10.1007/s11239-017-1508-y 28569368

[16] Gu X Li Y Chen S Yang X Liu F Li Y. Association of lipids with ischemic and hemorrhagic stroke: A prospective cohort study among 267 500 Chinese. Stroke (2019) 50:3376–84. doi: 10.1161/STROKEAHA.119.026402 31658904

[17] Dziedzic T Slowik A Gryz EA Szczudlik A. Lower serum triglyceride level is associated with increased stroke severity. Stroke (2004) 35:e151–152. doi: 10.1161/01.STR.0000128705.63891.67 15131316

[18] Pikija S Milevcic D Trkulja V Kidemet-Piskac S Pavlicek I Sokol N. Higher serum triglyceride level in patients with acute ischemic stroke is associated with lower infarct volume on CT brain scans. Eur Neurol (2006) 55:89–92. doi: 10.1159/000092780 16636555

[19] Rodriguez-Campello A Jimenez-Conde J Ois A Cuadrado-Godia E Giralt-Steinhauer E Vivanco RM. Sex-related differences in abdominal obesity impact on ischemic stroke risk. Eur J Neurol (2017) 24:397–403. doi: 10.1111/ene.13216 28032444

[20] Tang H Mo J Chen Z Xu J Wang A Dai L. Uric acid contributes to obesity-paradox of the outcome of ischemic stroke. Front Neurol (2019) 10:1279. doi: 10.3389/fneur.2019.01279 31866932 PMC6906190

[21] Xu J Wang A Meng X Jing J Wang Y Wang Y. Obesity-stroke paradox exists in insulin-resistant patients but not insulin sensitive patients. Stroke (2019) 50:1423–9. doi: 10.1161/STROKEAHA.118.023817 31043152

[22] Dehlendorff C Andersen KK Olsen TS. Body mass index and death by stroke: no obesity paradox. JAMA Neurol (2014) 71:978–84. doi: 10.1001/jamaneurol.2014.1017 24886975

[23] Tokunaga K Matsuzawa Y Ishikawa K Tarui S. A novel technique for the determination of body fat by computed tomography. Int J Obes (1983) 7:437–45. 6642855

[24] Ferland M Despres JP Tremblay A Pinault S Nadeau A Moorjani S. Assessment of adipose tissue distribution by computed axial tomography in obese women: association with body density and anthropometric measurements. Br J Nutr (1989) 61:139–48. doi: 10.1079/bjn19890104 2706220

[25] Ross R Aru J Freeman J Hudson R Janssen I. Abdominal adiposity and insulin resistance in obese men. Am J Physiol Endocrinol Metab (2002) 282:E657–663. doi: 10.1152/ajpendo.00469.2001 11832370

[26] Despres JP Lemieux I Bergeron J Pibarot P Mathieu P Larose E. Abdominal obesity and the metabolic syndrome: Contribution to global cardiometabolic risk. Arterioscler Thromb Vasc Biol (2008) 28:1039–49. doi: 10.1161/ATVBAHA.107.159228 18356555

[27] Lemieux I Pascot A Couillard C Lamarche B Tchernof A Almeras N. Hypertriglyceridemic waist: A marker of the atherogenic metabolic triad (hyperinsulinemia; hyperapolipoprotein b; small, dense LDL) in men? Circulation (2000) 102:179–84. doi: 10.1161/01.cir.102.2.179 10889128

[28] Sam S Haffner S Davidson MH D'Agostino RBSR Feinstein S Kondos G. Hypertriglyceridemic waist phenotype predicts increased visceral fat in subjects with type 2 diabetes. Diabetes Care (2009) 32:1916–20. doi: 10.2337/dc09-0412 PMC2752928 19592623

[29] Vaverkova H Karasek D Novotny D Halenka M Orsag J Slavik L. Hypertriglyceridemic waist - a simple clinical tool to detect cardiometabolic risk: comparison with harmonized definition of metabolic syndrome. Physiol Res (2015) 64:S385–394. doi: 10.33549/physiolres.933198 26680672

[30] Tian YM Ma N Jia XJ Lu Q. The "hyper-triglyceridemic waist phenotype" is a reliable marker for prediction of accumulation of abdominal visceral fat in Chinese adults. Eat Weight Disord (2020) 25:719–26. doi: 10.1007/s40519-019-00677-w 30982942

[31] Wang A Li Z Zhou Y Wang C Luo Y Liu X. Hypertriglyceridemic waist phenotype and risk of cardiovascular diseases in China: Results from the kailuan study. Int J Cardiol (2014) 174:106–9. doi: 10.1016/j.ijcard.2014.03.177 24745860

[32] Wang W Shen C Zhao H Tang W Yang S Li J. A prospective study of the hypertriglyceridemic waist phenotype and risk of incident ischemic stroke in a Chinese rural population. Acta Neurol Scand (2018) 138:156–62. doi: 10.1111/ane.12925 29574685

[33] Wang G Fang B Yu X Li Z. [Interpretation of 2018 guidelines for the early management of patients with acute ischemic stroke]. Zhonghua Wei Zhong Bing Ji Jiu Yi Xue (2018) 30:289–95. doi: 10.3760/cma.j.issn.2095-4352.2018.04.001 29663986

[34] Alberti KG Zimmet P Shaw J IDF Epidemiology Task Force Consensus Group. The metabolic syndrome–a new worldwide definition. Lancet (2005) 366:1059–62. doi: 10.1016/S0140-6736(05)67402-8 16182882

[35] Elkind MS Flint AC Sciacca RR Sacco RL. Lipid-lowering agent use at ischemic stroke onset is associated with decreased mortality. Neurology (2005) 65:253–8. doi: 10.1212/01.wnl.0000171746.63844.6a 16043795

[36] Adams HP Jr. Bendixen BH Kappelle LJ Biller J Love BB Gordon DL. Classification of subtype of acute ischemic stroke. definitions for use in a multicenter clinical trial. TOAST. trial of org 10172 in acute stroke treatment. Stroke (1993) 24:35–41. doi: 10.1161/01.str.24.1.35 7678184

[37] Ko Y Lee S Chung JW Han MK Park JM Kang K. MRI-Based algorithm for acute ischemic stroke subtype classification. J Stroke (2014) 16:161–72. doi: 10.5853/jos.2014.16.3.161 PMC4200592 25328874

[38] WHO. Guidelines for controlling and monitoring the tobacco epidemic. Geneva: Tobacco or Health Programme, WHO (1997). Available at: https://www.who.int/.

[39] Willenbring ML Massey SH Gardner MB. Helping patients who drink too much: An evidence-based guide for primary care clinicians. Am Fam Physician (2009) 80:44–50. doi: 10.1186/1471-2296-10-50 19621845

[40] Bureau of Disease P Control NHCoPsRoC National Center for Cardiovascular D Chinese Academy of Medical S Peking Union Medical College FH Chinese Center for C. National guideline for hypertension management in China (2019). Zhonghua Xin Xue Guan Bing Za Zhi (2020) 48:10–46. doi: 10.3760/cma.j.issn.0253-3758.2020.01.004 32008294

[41] Jia W Weng J Zhu D Ji L Lu J Zhou Z. Standards of medical care for type 2 diabetes in China 2019. Diabetes Metab Res Rev (2019) 35:e3158. doi: 10.1002/dmrr.3158 30908791

[42] Nutritional anaemias. report of a WHO scientific group. World Health Organ Tech Rep Ser (1968) 405:5–37. 4975372

[43] Weir CJ Sattar N Walters MR Lees KR. Low triglyceride, not low cholesterol concentration, independently predicts poor outcome following acute stroke. Cerebrovasc Dis (2003) 16:76–82. doi: 10.1159/000070119 12766366

[44] Tziomalos K Giampatzis V Bouziana SD Spanou M Kostaki S Papadopoulou M. Prognostic significance of major lipids in patients with acute ischemic stroke. Metab Brain Dis (2017) 32:395–400. doi: 10.1007/s11011-016-9924-9 27771869

[45] Ren Y Li H Wang X. Family income and nutrition-related health: Evidence from food consumption in China. Soc Sci Med (2019) 232:58–76. doi: 10.1016/j.socscimed.2019.04.016 31071477

[46] Lemieux I Poirier P Bergeron J Almeras N Lamarche B Cantin B. Hypertriglyceridemic waist: a useful screening phenotype in preventive cardiology? Can J Cardiol (2007) 23 Suppl B:23B–31B. doi: 10.1016/s0828-282x(07)71007-3 PMC2794461 17932584

[47] Fernandez-Garcia JC Munoz-Garach A Martinez-Gonzalez MA Salas-Salvado J Corella D Hernaez A. Association between lifestyle and hypertriglyceridemic waist phenotype in the PREDIMED-plus study. Obes (Silver Spring) (2020) 28:537–43. doi: 10.1002/oby.22728 32090511

[48] Yamashiro K Tanaka R Tanaka Y Miyamoto N Shimada Y Ueno Y. Visceral fat accumulation is associated with cerebral small vessel disease. Eur J Neurol (2014) 21:667–73. doi: 10.1111/ene.12374 24495037

[49] Kim KW Seo H Kwak MS Kim D. Visceral obesity is associated with white matter hyperintensity and lacunar infarct. Int J Obes (Lond) (2017) 41:683–8. doi: 10.1038/ijo.2017.13 28104915

[50] Pinto A Tuttolomondo A Di Raimondo D Fernandez P Licata G. Risk factors profile and clinical outcome of ischemic stroke patients admitted in a department of internal medicine and classified by TOAST classification. Int Angiol (2006) 25:261–7. 16878074

[51] Chen S Guo X Dong S Yu S Chen Y Zhang N. Association between the hypertriglyceridemic waist phenotype and hyperuricemia: A cross-sectional study. Clin Rheumatol (2017) 36:1111–9. doi: 10.1007/s10067-017-3559-z 28185015

[52] Zunker P Schick A Buschmann HC Georgiadis D Nabavi DG Edelmann M. Hyperinsulinism and cerebral microangiopathy. Stroke (1996) 27:219–23. doi: 10.1161/01.str.27.2.219 8571413

[53] Crosta F Occhiuzzi U Passalacqua G Occhiuzzi E Cimini A Grassi D. Association between the serum uric acid levels and lacunar infarcts in the elderly. J Mol Neurosci (2018) 65:385–90. doi: 10.1007/s12031-018-1096-0 29974380

